# Association of NT-proBNP and hs-cTnT with Imaging Markers of Diastolic Dysfunction and Focal Myocardial Fibrosis in Hypertrophic Cardiomyopathy

**DOI:** 10.3390/life12081241

**Published:** 2022-08-16

**Authors:** Céleste Chevalier, Miriam Wendner, Anna Suling, Ersin Cavus, Kai Muellerleile, Gunnar Lund, Paulus Kirchhof, Monica Patten

**Affiliations:** 1 Department of Cardiology, University Heart & Vascular Center Hamburg, University Medical Center Hamburg-Eppendorf, Martinistraße 52, 20246 Hamburg, Germany; 2 Institute of Medical Biometry and Epidemiology, Center for Experimental Medicine, University Medical Center Hamburg-Eppendorf, Christoph-Probst-Weg 1, 20246 Hamburg, Germany; 3 Deutsches Zentrum für Herz-Kreislauf-Forschung e.V. (German Center for Cardiovascular Research), Partner Site Hamburg/Kiel/Lübeck, 20246 Hamburg, Germany; 4 Department of Diagnostic and Interventional Radiology and Nuclear Medicine, University Medical Center Hamburg-Eppendorf, Martinistraße 52, 20246 Hamburg, Germany

**Keywords:** hypertrophic cardiomyopathy, biomarker, BNP, troponin, diastolic dysfunction, LGE

## Abstract

Serum biomarkers such as N-terminal prohormone of the brain natriuretic peptide (NT-proBNP) and cardiac troponins are elevated in patients with hypertrophic cardiomyopathy (HCM). At present, it is not clear if these markers are associated with distinct clinical alterations in HCM, such as left ventricular hypertrophy, outflow tract obstruction, myocardial fibrosis and/or diastolic dysfunction (DD), which are associated with adverse cardiovascular outcome. Here we evaluate the association of NT-proBNP and high sensitivity cardiac troponin T (hs-cTnT) to a variety of cardiac imaging parameters in HCM patients in a multivariable regression analysis. This retrospective cross-sectional study included 366 HCM patients who underwent transthoracic echocardiography (TTE), 218 of whom also obtained cardiovascular magnetic resonance (CMR) to assess focal myocardial fibrosis by LGE. Multivariable regression analyses revealed the strongest association of the DD parameters E/E′ mean and E/E′ septal with NT-proBNP (b = 0.06, 95%-CI [0.05–0.07], *p* < 0.001, R^2^ = 0.28; b = 0.08, 95%-CI [0.06–0.1], *p* < 0.001, R^2^ = 0.25) and LGE size showed the strongest association with hs-cTnT (b = 0.20, 95%-CI [0.15–0.24], *p* < 0.001, R^2^ = 0.28). This study indicates that NT-proBNP and hs-cTnT are associated with structural and functional alterations in HCM. NT-proBNP is a stronger predictor for DD, while hs-cTnT is associated with the extent of focal myocardial fibrosis. Both biomarkers might be useful in the diagnostic procedure in addition to imaging parameters.

## 1. Introduction

Hypertrophic cardiomyopathy (HCM) is characterized by an inappropriate hypertrophy of the left ventricle with hypertrophic and disarrayed myocytes as a result of mutations in genes encoding for sarcomere proteins [1]. Previous studies have frequently found elevated serum levels of brain natriuretic peptide (BNP) and high sensitivity cardiac troponin T (hs-cTnT) in HCM patients [2,3,4,5,6,7,8,9,10,11]. For both biomarkers, studies also reported associations with characteristic features of HCM, such as left ventricular hypertrophy (LVH) [3,7,9,10,11,12,13,14,15,16,17], left ventricular outflow tract (LVOT) gradient [9,10,11,18], imaging parameters of diastolic dysfunction (DD) [5,9,10,14,15,16,19], and focal myocardial fibrosis assessed by late gadolinium enhancement (LGE) [6,8,9,11,12,17,20]. These structural and functional changes [21,22,23,24,25,26,27,28,29,30] are common in HCM and related to adverse cardiovascular outcome [21,22,25,26,27,31,32,33]. The non-invasive diagnosis of LVOT obstruction, DD [34], and myocardial fibrosis [35] are based on imaging diagnostics, transthoracic echocardiography (TTE), and cardiovascular magnetic resonance (CMR), respectively.

TTE and CMR are generally accepted and established techniques but also entail limitations due to, for instance, contraindications or availability in the case of CMR, or operator-dependency in the case of TTE. Circulating biomarkers on the other hand are easy to determine as objective parameters in the diagnostic procedure.

Here, we aim to evaluate the association of N-terminal prohormone of the brain natriuretic peptide (NT-proBNP) and hs-cTnT with characteristic disease markers of HCM, by applying an overall comprehensive assessment that allows for the analysis of both biomarkers and multiple imaging parameters.

## 2. Materials and Methods

### 2.1. Study Population

A total of 366 HCM patients were included in this retrospective cross-sectional study. Participants were enrolled during routine visits at the outpatient clinic of the University Heart & Vascular Center Hamburg between January 2011 and December 2019. HCM was defined by a maximum wall thickness (MWT) of ≥15 mm in one or more left ventricular (LV) myocardial segments in the absence of abnormal loading conditions according to current guidelines [36]. Patients with a characteristic genetic disposition (pathogenic class 4 or 5 mutation) were also included. Exclusion criteria were pregnancy and an estimated glomerular filtration rate (eGFR) < 30 mL/min/1.73 m^2^. A medical history of coronary artery disease (CAD), diabetes mellitus, and arterial hypertension was confirmed by self-report or the use of corresponding medication. The diagnosis of atrial fibrillation (AF) was established by a positive history and electrocardiogram (ECG) documentation within the last five years prior to examination. Cardiac symptoms were classified according to the New York Heart Association (NYHA) classification. All participants received a 12-lead surface ECG, a TTE, and had routine blood work done, including measurement of hs-cTnT and NT-proBNP. Serum levels of NT-proBNP were assessed by the Atellica^®^ IM NT-proBNP assay (Siemens Healthcare, Erlangen, Germany). For the measurement of serum hs-cTnT levels, the Elecsys Troponin T hs STAT assay (Roche Diagnostics, Risch-Rotkreuz, Switzerland) was performed. In addition, 218 patients underwent CMR. The study was conducted in compliance with the principles outlined in the Declaration of Helsinki and was approved by the local ethics committee (Ethikkommission der Ärztekammer Hamburg, Nr.: PV4056). All study participants gave their written informed consent.

### 2.2. Echocardiography

All study subjects underwent a comprehensive TTE examination (Philips iE33 system, Philips Healthcare, Best, Netherlands) including M-mode, two-dimensional (2D), pulsed and continuous-wave Doppler, and tissue Doppler imaging (TDI). Structural and functional imaging parameters were measured according to current recommendations of the American Society of Echocardiography, and DD was classified according to current guidelines [34]. Resting and provoked (using the Valsalva maneuver) LVOT flow gradients were assessed, and patients were divided into having non-obstructive (HNOCM; resting LVOT gradient < 30 mmHg), latent obstructive (HLOCM; resting LVOT gradient < 30 mmHg, provoked LVOT gradient ≥ 50 mmHg), and obstructive hypertrophic cardiomyopathy (HOCM; resting LVOT gradient ≥ 30 mmHg) according to current guidelines [36]. Images were analyzed using the commercially available software Syngo Dynamics (Siemens Healthcare, Erlangen, Germany).

### 2.3. CMR Protocol and Analysis

CMR was performed on a 1.5-T scanner (Achieva, Philips Healthcare, Best, The Netherlands). The imaging protocol included cine imaging and LGE imaging. Standard retrospectively gated steady state free precession (SSFP) cine sequences were acquired in short-axis slices covering the LV and in long-axis view (four-chamber (4CH), two-chamber (2CH), and three-chamber (3CH) view). Typical imaging parameters were as follows: voxel size 1.40 × 1.40 × 8 mm^3^, echo time = 1.60 ms, time to repetition = 3.20 ms, flip angle = 60°, parallel acquisition technique = SENSE. LGE images were acquired using a standard phase-sensitive inversion recovery (PSIR) sequence at least ten minutes after bolus injection of gadoterate meglumine (Dotarem, Guerbet, Sulzbach, Germany) in three long-axis orientations (2CH view, 3CH view, 4CH view) and a stack of short-axis slices. Typical imaging parameters were as follows: voxel size 0.98 × 0.98 × 8 mm^3^, echo time = 2.39 ms, time to repetition = 4.97 ms, flip angle = 15°. The presence and extent of LGE was assessed by using the commercially available software cvi42 (Circle Cardiovascular Imaging Inc., Calgary, AB, Canada) and by applying the standard deviation-based (3-SD) method as recommended [37]. The amount of LGE was given in g and % of LV mass.

### 2.4. Statistical Analysis

Statistical analysis was performed using IBM SPSS Statistics (Version 28.0, Statistical Package for the Social Sciences, International Business Machines, Inc., Armonk, New York, NY, USA). Continuous data are given as mean and standard deviation (SD) or as median and interquartile range (Q1 and Q3), according to the visual evaluation of the normality assumption. Categorical data are given as frequencies and percentages. Outliers were identified via evaluation of the standardized residues and included when measurement errors could be excluded. To determine the association of NT-proBNP and hs-cTnT with echocardiographic and CMR imaging markers, separate linear regression analyses were performed with NT-proBNP or hs-cTnT as the independent variable. The dependent variables were as follows: SCD (sudden cardiac death) Risk Score, LA (left atrial) diameter, resting and provoked LVOT gradient, SW (septal wall) thickness, mean, septal and lateral E/E′ (peak early transmitral filling velocity/early mitral annulus velocity), septal and lateral IVRT (isovolumetric relaxation time), and LGE size. Resting and provoked LVOT gradient were logarithmized. Only continuous variables were examined. All models were adjusted for age, sex, body mass index (BMI), AF, eGFR, and arterial hypertension. Slopes (b) are presented with 95% confidence intervals (CI). Histograms of residuals were checked for deviations of the normality assumption. A *p*-value < 0.05 was considered statistically significant.

## 3. Results

### 3.1. Baseline Characteristics

Detailed clinical characteristics of the study population are presented in Table 1. The majority of patients reported symptoms of heart failure; 112 patients (31%) were asymptomatic (NYHA functional class I), 154 patients (42%) reported symptoms with moderate exertion (NYHA functional class II), and 100 patients (27%) reported symptoms with minimal exertion (≥NYHA functional class III). A total of 146 patients (41%) had elevated hs-cTnT levels > 14 pg/mL, of which 21 patients (14%) had a reduced eGFR of 30–50 mL/min/1.73 m^2^. A total of 311 patients (86%) showed raised NT-proBNP levels > 125 ng/L, of which 25 (8%) had a reduced eGFR. Table 2 shows the echocardiography and CMR parameters of the study population. The majority of patients (92%) presented with normal left ventricular ejection fraction (LVEF). DD was detected in 256 patients (71%), with 137 (38%) being classified as moderate to severe. In 17 patients (5%) SW thickness was <15 mm. They met the inclusion criteria by having either an apical HCM (*n* = 1) or a pathogenic class 5 mutation. CMR was performed in 218 patients (59%). Of these, 21 patients had to be excluded from analysis due to missing (*n* = 9) or inadequate image quality (*n* = 14) of PSIR sequences. Three patients had to be excluded from analysis due to concomitant LGE in the context of other myocardial diseases. LGE was identified in 150 HCM patients (78%) with sufficient CMR. Median LGE extent of total myocardial mass was 3.7 [IQR 0.8–6.9] % of LV mass. CMR was not conducted in patients due to claustrophobia, metal implants, or non-MR compatible cardiac pacemaker or implantable cardioverter defibrillator *(n* = 43). A total of 11 patients received an ICD early after MRI. Externally performed MRI studies were not included.

### 3.2. Regression Analysis

#### 3.2.1. Association of NT-proBNP with Echocardiographic Parameters of Diastolic Dysfunction

**Figure 1:**
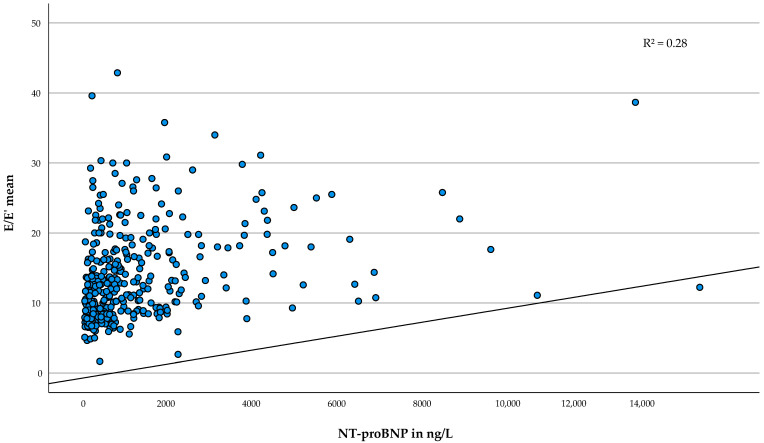
Association of serum NT-proBNP with E/E′ mean. Scatterplot of E/E′ mean and NT-proBNP in ng/L. The regression line is adjusted according to the multivariable model. Abbreviations: E = peak early transmitral filling velocity, E′ = early mitral annulus velocity, NT-proBNP = N-terminal prohormone of the brain natriuretic peptide, R^2^ = adjusted coefficient of determination.

As shown in Table 3, NT-proBNP was associated with all considered variables except with the SCD Risk Score (*p* = 0.092) and provoked LVOT flow gradient (*p* = 0.101). Judged by the R^2^, the strongest association was found for E/E′ mean (b = 0.06, 95%-CI [0.05–0.07], *p* < 0.001, R^2^ = 0.28) and E/E′ septal (b = 0.08, 95%-CI [0.06–0.10], *p* < 0.001, R^2^ = 0.25). An increase in NT-proBNP by 50 ng/L was associated with an increase in E/E′ mean by 0.06 or in E/E′ septal by 0.08. Figure 1 depicts the association of serum NT-proBNP with E/E′ mean.

#### 3.2.2. Association of hs-cTnT with Focal Myocardial Fibrosis

**Figure 2:**
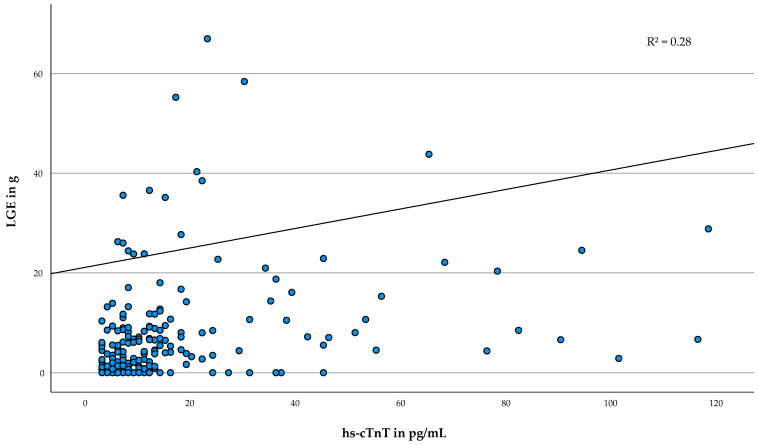
Association of serum hs-cTnT with the amount of LGE. Scatterplot of LGE size in g and hs-cTnT in pg/mL. The regression line is adjusted according to the multivariable model. Abbreviations: hs-cTnT = high sensitivity cardiac troponin T, LGE = late gadolinium enhancement, R^2^ = adjusted coefficient of determination.

The results of the multivariable regression analysis to evaluate the association between hs-cTnT and the respective imaging markers are presented in Table 4. Hs-cTnT was associated with all included parameters except for E/E′ lateral (*p* = 0.161) and the logarithmized resting and provoked LVOT gradient (*p* = 0.195; *p* = 0.616). Overall, LGE size showed the strongest association with hs-cTnT (b = 0.20, 95%-CI [0.15–0.24], *p* < 0.001, R^2^ = 0.28). Figure 2 illustrates the association of serum hs-cTnT with the amount of LGE.

## 4. Discussion

We evaluated the association of NT-proBNP and hs-cTnT with different disease features of HCM. Both biomarkers were associated with a variety of characteristic markers such as LVH, DD, and focal fibrosis. However, our data indicate the strongest association of elevated NT-proBNP levels with echocardiographic parameters of DD and hs-cTnT levels with LGE size measured by CMR.

### 4.1. NT-proBNP and Diastolic Dysfunction in HCM

The neurohormone NT-proBNP is released in response to LV wall stress due to ventricular volume or pressure overload [38,39]. It is a well-established biomarker for heart failure and part of the gold standard examination in patients with suspected heart failure recommended by the 2021 ESC guidelines on heart failure, even with preserved ejection fraction (HFpEF) [40].

The majority of HCM patients exhibit some level of DD, and most of them suffer from HFpEF in the course of disease progression [28,41,42,43,44]. DD represents a key factor in the pathophysiology of HCM. The etiology of DD in HCM includes several aspects, such as alterations in LV morphology and tissue composition, myocardial ischemia, as well as changes on a cellular and molecular level [30,45]. They ultimately lead to myocardial stiffness and reduced compliance with an increase in LV diastolic stress [28,29]. DD has been associated with poor prognosis in HCM [31,33]. The fact that NT-proBNP is mostly predictive of endpoints, including heart failure in HCM [18], further emphasizes an association of BNP with DD. Patients with HCM characteristically have normal LVEF. Only a small percentage (2–9%) of end-stage patients suffer from significant LVEF reduction [41,42,43,44], indicating that a rise in NT-proBNP values is most likely connected to a worsening in diastolic function.

In line with our findings demonstrating elevated NT-proBNP levels > 125 pg/mL in 86% of HCM patients, several studies have reported abnormal proBNP or NT-proBNP levels in HCM [2,4,5,9,10]. It has been shown that NT-proBNP is an independent predictor of mortality in HCM [46], and that HCM patients with an abnormal NT-proBNP had a seven-fold increase in the risk of death or the need for transplantation [18], and a higher rate of cardiovascular events [47].

BNP levels have been found to be associated with multiple HCM disease markers such as NYHA functional class, LVEF, LVOT obstruction, LVH [9,10,18,48,49], LGE [9,50], as well as parameters of DD [5,10,14,15,19]. Kim et al. showed higher NT-proBNP levels in asymptomatic to mildly symptomatic HCM patients with DD compared with patients without DD. Correlation analyses of NT-proBNP and markers of echocardiographic parameters of DD point to an association as well [10,14,15,51].

Although we detected only a marginally stronger association of NT-proBNP with septal E/E′ than with lateral E/E′ in our cohort, this was supported by the findings of Nakamura et al. showing a slightly stronger correlation of BNP values with septal E/E′ than with lateral E/E′ [51]. These findings might be related to a pronounced relaxation abnormality of the septal wall in HCM patients as reported by Voigt et al., observing a higher septal to lateral IVRT ratio in the mitral annulus TDI in HCM patients compared to other entities of LVH [52].

However, Binder et al. could only show a weak correlation of BNP with calculated wall stress as a marker for DD [49]. Overall, these findings support our results of an association of NT-proBNP with echocardiographic markers of DD in HCM, with the strongest association observed for E/E′ mean.

Moreover, we found an association of NT-proBNP with SW thickness, the predominant location of LVH [53], which has also been reported in previous studies [9,48]. A link between LV hypertrophy and DD through a smaller LV cavity, impaired LV relaxation, and increased enddiastolic pressure seems plausible. Yet, in our cohort LVH does not seem to be the only contributor as the association of NT-proBNP with SW thickness was less strong than the association of NT-proBNP with E/E′ mean.

HCM patients with LVOT obstruction have been reported to have particularly high levels of BNP [54], and studies have demonstrated a strong correlation of BNP or NT-proBNP with the LVOT gradient [10,55,56]. In our analyses, however, the relation of NT-proBNP with resting LVOT gradient was less prominent compared with other disease markers.

### 4.2. Hs-cTnT and Fibrosis in HCM

**Figure 3:**
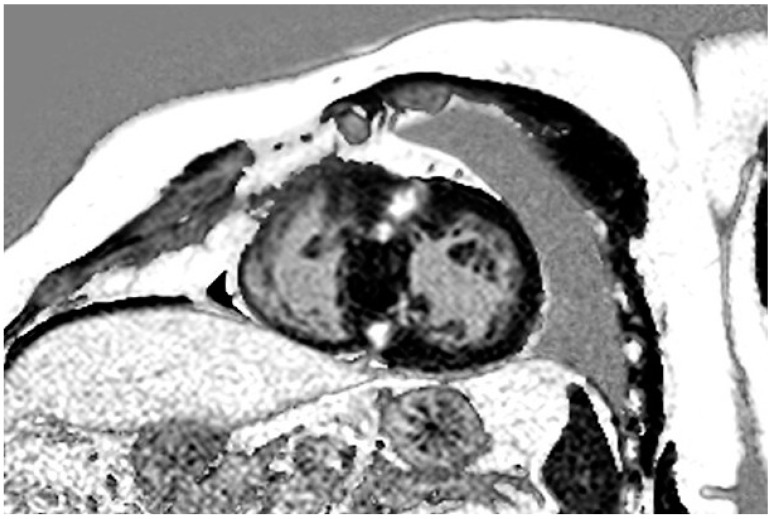
LGE in a patient with HCM. LGE in a typical patchy mid-wall pattern in hypertrophic segments and at RV insertion points. Abbreviations: HCM = hypertrophic cardiomyopathy, LGE = late gadolinium enhancement, RV = right ventricular.

We detected focal myocardial fibrosis on LGE imaging in 150 HCM patients (77%), which is consistent with previously published studies that have reported LGE in 42 to 72% of cases [11,25,26,27,32,57]. Figure 3 shows a typical LGE distribution pattern in an HCM patient. A meta-analysis by Weng et al. comprising five studies showed that LGE presence and LGE extent were associated with cardiovascular and all-cause mortality in HCM [27]. LGE has been described as risk marker for sudden cardiac death (SCD) [26,27,57], as well as for LV dysfunction and progression to heart failure [26,58]. An accurate assessment of myocardial fibrosis therefore seems crucial in the individual risk assessment of HCM patients, in particular as LGE seems to be present also in asymptomatic or mildly symptomatic individuals [25], and is not necessarily associated with symptom severity [57].

CMR is a reliable alternative to endomyocardial biopsy in identifying myocardial fibrosis [20], but nevertheless has some limitations. Higher costs, limited availability, and contraindications limit the broad use of CMR.

Hs-cTnT is already an established biomarker for the detection of myocardial injury with high sensitivity and specificity [59,60]. An association with cardiovascular events was found already for a hs-cTnT cut-off value of 14 pg/mL. The risk increased with the degree of elevated hs-cTnT values [7].

In HCM, several studies have described elevated troponin levels with a prevalence of 40 to 55% [3,6,8,11,13]. We similarly found an abnormal hs-cTnT value of >14 pg/mL in 41% of our patients. Raised cTn levels have been associated with clinical parameters of HCM disease severity such as heart failure symptoms, AF, and syncope [7] as well as structural and functional parameters including MWT, left ventricular myocardial mass (LVM), LV systolic and diastolic dysfunction, LA diameter, and LVOT gradient [3,7,8,11,47,61]. Furthermore, cTn has been determined as a risk marker and independent predictor of cardiovascular events [7,47,62].

The cause of myocardial cell death and troponin release in HCM still remains unclear. Possible underlying mechanisms include myocardial ischemia due to increased oxygen demand, and reduced capillary density of the hypertrophic myocardium as well as overdistension of sarcomeres due to reduced compliance and myocyte disarray [3,7,63,64,65].

A higher frequency of myocardial cell death is accompanied by myocardial replacement fibrosis, which supports a potential relation of cTn levels and fibrosis. Previous studies have reported an association of hs-cTnT levels with LGE as a marker for fibrosis in HCM, similar to our results. In addition, several studies have demonstrated elevated serum levels of cTnI in LGE-positive HCM patients compared to LGE-negative patients [6,8,11,17]. Besides the mere presence of LGE, some have found an additional association of LGE extent with troponin levels [66,67], which is in line with our observations, while others have not [6,12]. In contrast to these results and our observation, a study by Cramer et al. did not show a correlation of cTn with LGE [3]. Li et al., despite recording higher serum levels of cTnI in LGE positive patients, could also not identify cTnI as predictor of the presence of LGE in a multivariate analysis [17].

The fact that LGE is often found in hypertrophic segments [17,68] and associated with MWT and greater hypertrophy [3,6,11,17], similarly to cTn [8,61,67], further strengthens the point of a possible association of LGE and cTn. It seems plausible that areas of inadequate hypertrophy exhibit lower perfusion and higher wall stress, with consequently higher rates of cell death and fibrosis and presumably higher cTn release [17]. Yet, cTn has also been determined as an independent predictor of LGE after adjusting for MWT and LVMI [11], indicating that cell death, fibrosis, and cTn release are not solely linked to hypertrophy. Our results strengthen this point as hs-cTnT was also significantly associated with SW thickness, but to a lesser extent than the association of hs-cTnT with LGE.

### 4.3. Overlap between Associations

Finally, in line with our observations, previous studies have also reported a relation between cTn and markers of DD [8,13,16], and a relation of BNP with LGE [6,9,11,12,17]. Our analyses allowed for a comprehensive assessment including both biomarkers and both imaging parameters. Fibrosis leads to increased ventricular stiffness with reduced compliance and consequently increased ventricular wall stress, which might result in the release of BNP [4,9,69]. The fact that BNP has been found to be produced by fibroblasts [70] also supports a relation to fibrosis. In turn, DD and increased LV end-diastolic pressure can promote myocardial cell death and account for troponin release, e.g., due to decreased subendocardial perfusion and ischemia [13,71]. Hessel et al. have also described stretch-related mechanisms of troponin release without actual cell death, which could be relevant to DD as well [13,65].

Overall, our results show associations of the blood biomarkers NT-proBNP and hs-cTnT with structural and functional alterations in HCM. Hence, these biomarkers might be useful in a comprehensive assessment of patients next to diagnostic imaging. Further investigations are needed to evaluate the reliability of these blood biomarkers for early assessment of fibrosis and DD in HCM patients.

## 5. Conclusions

We found associations of NT-proBNP and hs-cTnT with structural and functional alterations in HCM patients. As NT-proBNP is more closely associated with DD, while hs-cTnT is associated with the size of focal myocardial fibrosis, it might be useful to incorporate both biomarkers in the diagnostic procedure next to diagnostic imaging. After further analyses, the biomarkers might also be helpful parameters for the identification of a more precise phenotype and for potential guidance of specific therapeutic strategies in these patients.

## 6. Limitations

We acknowledge the following limitations: First, there are limitations due to the cross-sectional study design. Our results should therefore be confirmed in a longitudinal study including serial measurements. Secondly, blood biomarkers are prone to several influencing factors, which might impact their predictive value. However, this study demonstrates significant associations of circulating and imaging biomarkers and provides supportive evidence for future analyses. Thirdly, LGE imaging was not available for all patients, and we did not assess T1 mapping. We could therefore not evaluate diffuse fibrosis next to LGE as a marker for focal fibrosis.

## Figures and Tables

**Table 1:** Clinical characteristics.

Clinical Parameters (Unit)	Median (IQR)/*n*-Number (Percentage)/Mean (±SD)	Range	*N*
Age (years)	54 (±16)	16–87	366
Sex (males, *n*)	206 (56%)		366
BMI (kg/m^2^)	27 (±5)	14–49	361
Arterial hypertension (*n*)	170 (47%)		365
Diabetes mellitus (*n*)	40 (11%)		364
AF (*n*)	104 (28%)		366
Ventricular tachycardia (medical history, *n*)	82 (22%)		365
Sudden cardiac death (family history, *n*)	92 (25%)		361
Syncope (*n*)	71 (20%)		359
SCD Risk Score	3 (2–5)	0.72–22.23	336
<4% (*n*)	221 (66%)		
≥4, <6% (*n*)	56 (17%)		
≥6% (*n*)	59 (17%)		
ICD (*n*)	54 (15%)		366
CAD (*n*)	59 (16%)		365
Myocardial infarction (*n*)	8 (2%)		366
NYHA functional class (*n*)			366
I	112 (31%)		
II	154 (42%)		
III	99 (27%)		
IV	1 (<1%)		
eGFR (mL/min/1.73 m^2^)	80 (±24)	31–200	364
eGFR of 30–50 mL/min/1.73 m^2^ (*n*)	26 (7%)		
Hs-cTnT (pg/mL)	12 (7–21)	3–416	358
>14 pg/mL (*n*)	146 (41%)		
NT-proBNP (ng/L)	663 (234–1534)	5–31,505	362
>125 ng/L (*n*)	311 (86%)		

Values for continuous data are given as mean and standard deviation or as median and interquartile range (Q1 and Q3) according to the visual evaluation of the normality assumption. Values for categorical data are given as counts and percentage of total column number. Abbreviations: AF = atrial fibrillation, BMI = body mass index, CAD = coronary artery disease, eGFR = estimated glomerular filtration rate, NYHA = New York Heart Association, SCD = sudden cardiac death.

**Table 2:** Echocardiography and CMR data.

Imaging Parameters (Unit)	Median (IQR)/*n*-Number (Percentage)/Mean (±SD)	Range	*N*
Echocardiography			
LVEF (*n*)			366
Normal (>50%)	336 (92%)		
Mildly reduced (41–49%)	20 (5%)		
Moderately reduced (30–40%)	4 (1%)		
Severely reduced (<30%)	6 (2%)		
SW thickness (mm)	21 (±5)	9–48	366
LW thickness (mm)	14 (±3)	5–32	347
LA diameter (mm)	46 (±10)	24–97	365
Resting LVOT flow gradient (mmHg)	11 (5–30)	2–210	365
Provoked LVOT flow gradient (mmHg)	20 (8–50)	1–234	288
Obstruction (*n*)			365
HNOCM	245 (67%)		
HLOCM	28 (8%)		
HOCM	92 (25%)		
Diastolic Dysfunction (*n*)			361
No DD	105 (29%)		
Mild DD	119 (33%)		
Moderate or severe DD	137 (38%)		
IVRT septal (ms)	132 (±40)	60–363	351
IVRT lateral (ms)	107 (±33)	46–299	353
E/A	1.2 (0.8–1.6)	0.1–4.6	347
E/E′ mean	12.3 (9.1–17.6)	1.7–57.2	355
E/E′ septal	15.0 (11.5–21.9)	5.1–71.5	357
E/E′ lateral	10.4 (7.7–15.6)	0.9–47.7	357
**Cardiovascular Magnetic Resonance Imaging**			
LGE (*n*)	150 (78%)		193
LGE size (g)	4.7 (0.9–9.7)	0–169.3	193
LGE size (% of LV mass)	3.7 (0.8–6.9)	0–43.9	193

Values for continuous data are given as mean and standard deviation or as median and interquartile range (Q1 and Q3) according to the visual evaluation of the normality assumption. Values for categorical data are given as counts and percentage of total column number. Abbreviations: A = peak late transmitral filling velocity, DD = diastolic dysfunction, E = peak early transmitral filling velocity, E′ = early mitral annulus velocity, HCM = hypertrophic cardiomyopathy, HLOCM = latent obstructive HCM, HNOCM = non-obstructive HCM, HOCM = obstructive HCM, IVRT = isovolumetric relaxation time, LA = left atrial, LGE = late gadolinium enhancement, LVEF = left ventricular ejection fraction, LV mass = Left ventricular mass, LVOT = left ventricular outflow tract, LW = lateral wall, SW = septal wall. Note: A total of 21 patients had to be excluded from analysis due to missing (*n* = 9) or inadequate quality of PSIR sequences (*n* = 13). A total of 3 patients had to be excluded due to concomitant myocardial disease with competing LGE.

**Table 3:** Regression analysis of imaging markers of diastolic dysfunction and myocardial fibrosis by NT-proBNP (at intervals of 50 ng/L).

Variables (Unit)	b	95 % CI	*p*-Value	*N*	R^2^
SCD Risk Score (%)	0.006	−0.001–0.012	0.095	329	0.11
LA diameter (mm)	0.026	0.006–0.046	0.010	355	0.18
Resting LVOT flow gradient (ln *)	1.005	1.003–1.007	<0.001	355	0.13
Provoked LVOT flow gradient (ln *)	1.003	0.999–1.006	0.101	278	0.11
E/E′ mean	0.058	0.045–0.072	<0.001	347	0.28
E/E′ septal	0.080	0.060–0.100	<0.001	349	0.25
E/E′ lateral	0.049	0.036–0.062	<0.001	349	0.22
IVRT septal	0.165	0.078–0.252	<0.001	343	0.09
IVRT lateral	0.218	0.146–0.290	<0.001	345	0.12
SW thickness (mm)	0.035	0.024–0.047	<0.001	356	0.11
LGE size (g)	0.123	0.068–0.179	<0.001	190	0.11

b = unstandardized regression coefficient, CI = confidence interval, R^2^ = adjusted coefficient of determination. * For logarithmized parameters the exponentiated regression estimates and confidence intervals are presented. Abbreviations: E = peak early transmitral filling velocity, E′ = early mitral annulus velocity, IVRT = isovolumetric relaxation time, LA = left atrial, LGE = late gadolinium enhancement, ln = logarithmized, LVOT = left ventricular outflow tract, SCD = sudden cardiac death, SW = septal wall.

**Table 4:** Regression analysis of imaging markers of diastolic dysfunction and myocardial fibrosis by hs-cTnT.

Variables (Unit)	b	95% CI	*p*-Value	*N*	R^2^
SCD Risk Score (%)	0.016	0.005–0.027	0.004	325	0.13
LA diameter (mm)	0.043	0.016–0.071	0.002	351	0.19
Resting LVOT flow gradient (ln *)	1.002	0.999–1.005	0.195	351	0.08
Provoked LVOT flow gradient (ln *)	1.001	0.997–1.004	0.616	274	0.11
E/E′ mean	0.022	0.001–0.042	0.041	342	0.14
E/E′ septal	0.037	0.007–0.067	0.016	344	0.12
E/E′ lateral	0.014	−0.006–0.034	0.161	344	0.12
IVRT septal	0.233	0.114–0.352	<0.001	338	0.09
IVRT lateral	0.180	0.078–0.281	<0.001	340	0.06
SW thickness (mm)	0.049	0.033–0.066	<0.001	352	0.11
LGE size (g)	0.196	0.147–0.244	<0.001	186	0.28

b = unstandardized regression coefficient, CI = confidence interval, R^2^ = adjusted coefficient of determination. * For logarithmized parameters the exponentiated regression estimates and confidence intervals are presented. Abbreviations: E = peak early transmitral filling velocity, E′ = early mitral annulus velocity, IVRT = isovolumetric relaxation time, LA = left atrial, LGE = late gadolinium enhancement, ln = logarithmized, LVOT = left ventricular outflow tract, SCD = sudden cardiac death, SW = septal wall.
